# Developing a Next-of-Kin Involvement Guide in Cancer Care—Results From a Consensus Process

**DOI:** 10.1097/NCC.0000000000000869

**Published:** 2020-08-03

**Authors:** Inger J. Bergerød, Geir S. Braut, Birte Fagerdal, Bjørnar Gilje, Siri Wiig

**Affiliations:** Author Affiliations: Stavanger University Hospital (Ms Bergerød and Drs Braut and Gilje); SHARE—Centre for Resilience in Healthcare, Faculty of Health Sciences, University of Stavanger (Ms Bergerød and Dr Wiig); Stavanger Unversity Hospital, Department of Hematology and Oncology (Ms Bergerød and Dr Gilje); and Haukeland University Hospital, Bergen, Norway (Ms Fagerdal).

**Keywords:** Cancer nursing, Consensus methods, Guide, Hospitals, Involvement, Next-of-kin, Nominal group technique

## Abstract

**Background:**

In hospital cancer care, there is no set standard for next-of-kin involvement in improving the quality of care and patient safety. There is therefore a growing need for tools and methods that can guide this complex area.

**Objective:**

The aim of this study was to present the results from a consensus-based participatory process of designing a guide for next-of-kin involvement in hospital cancer care.

**Method:**

A consensus process based on a modified Nominal group technique was applied with 20 stakeholder participants from 2 Norwegian university hospitals.

**Result:**

The participants agreed on the 5 most important priorities for hospital cancer care services when involving next-of-kin. The results showed that next-of-kin stakeholders, when proactively involved, are important resources for the patient and healthcare professionals in terms of contribution to quality and safety in hospitals. Suggested means of involving next-of-kin were closer interaction with external support bodies, integration in clinical pathways, adjusted information, and training healthcare professionals.

**Conclusion:**

In this study, we identified topics and elements to include in a next-of-kin involvement guide to support quality and safety in hospital cancer care. The study raises awareness of the complex area of next-of-kin involvement and contributes with theory development and knowledge translation in an involvement guide tailored for use by healthcare professionals and managers in everyday clinical practice.

**Implications for Practice:**

Service providers can use the guide to formulate intentions and make decisions with suggestions and priorities or as a reflexive tool for organizational improvement.

## Background

Over the last decade there have been many attempts to improve quality and safety for patients in healthcare services; however, hospitals still report poor patient outcomes.^1,2^ Next-of-kin and family caregivers are important collaborative partners in keeping patients safe both in hospitals and at home.^3–5^ They are, however, seldom considered equal partners in the medical team around the patient despite taking on many important care tasks in different parts of the cancer care trajectory.^6–8^ Consequently, next-of-kin may feel overburdened and stressed.^9–11^ In hospital cancer care, there is no set standard associated with next-of-kin involvement in general treatment or in relation to improving cancer service quality and safety.^12^ Next-of-kin involvement is seldom directly related to quality and safety, and research on this topic is rare.^12,13^ Previous research has identified a need for tools and methods to guide the complex area of next-of-kin involvement in general and in relation to the context of the involvement (eg, cancer care, pediatrics, geriatric care, intensive care).^8,14,15^ Such a development should incorporate a multistakeholder perspective that includes healthcare professionals, patients, and next-of-kin.^16^ Our study therefore takes this perspective.

Consensus methods are widely used in healthcare research to aid decision making, problem solving and idea generation.^17–19^ Consensus methods often gather experts in a field, such as oncologists or nurses to determine consensus on a given topic. There is, however, a lack of research on how to gather stakeholders across hospitals with a combined multidisciplinary, patient, and stakeholder perspective to arrive at a consensus on a topic from a group of representatives with diverse backgrounds and roles.^12^ Some topics, such as how to guide next-of-kin involvement in cancer care, as in our study, requires a broad representation of stakeholders to incorporate different perspectives in a consensus process and reach an agreement on the way forward (in other words, to cocreate).^20,21^

Consensus methods have multifaceted challenges. There are many potential practical obstacles, such as funding, time, organization and geography, when establishing an arena for the sharing of ideas and learning.^18^ Consequently, the method may fail without careful attention to the cocreation of knowledge between stakeholder groups and researchers.^22–24^

### Aim and Research Questions

With this in mind, we invited stakeholder representatives from 2 Norwegian hospitals to join a panel where we used a modified nominal group technique (NGT).^25^

The overarching research problem for the panel was as follows: What topics and elements should be included in a next-of-kin involvement guide to support quality and safety in hospital cancer care?

The following research questions guided the consensus process:

1) What can we learn from next-of-kin experiences with hospital cancer care?
2) How can next-of-kin experiences be valued more systematically to improve the quality and safety of cancer care?
3) What methods or tools are appropriate for collecting experiences and for next-of-kin involvement locally, regionally, and nationally?

Based on the consensus technique, we developed a guide for use in hospital cancer care to increase the focus on involvement and take advantage of the experiences of cancer patients’ next-of-kin. The aim of this article is to present the results from the consensus process and to produce a guide for next-of-kin involvement in hospital cancer care.

### Study Design and Setting

**Figure 1:**
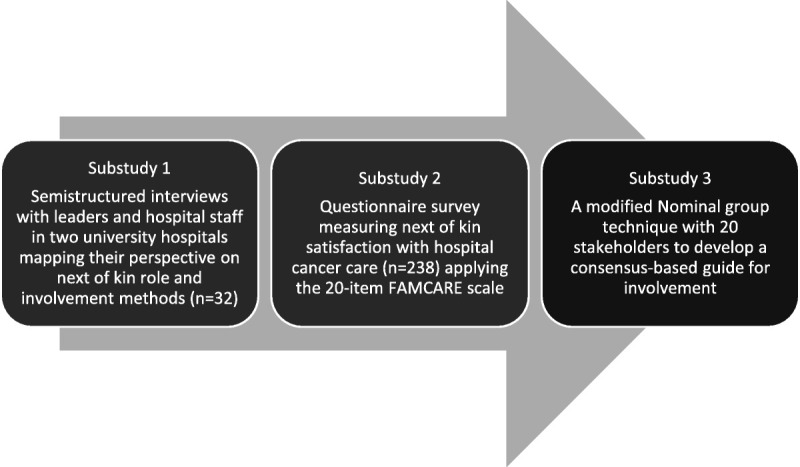
Overview of the project.

This article is part of a mixed-method project with a convergent design.^26^ The design consists of 3 substudies that explore quality and safety in hospital cancer care in 2 Norwegian university hospitals (Figure 1).

Substudy 1 was a qualitative mapping of next-of-kin involvement and involvement methods in cancer care services in the 2 hospitals. This was an in-depth study of managers’ and hospital staffs’ perspectives. The study resulted in 2 published articles.^8,27^ Substudy 2 was a quantitative measurement of next-of-kin satisfaction with cancer care services in the 2 hospitals and resulted in 1 published article.^15^ Substudy 3, reported here, is a consensus process (using the NGT) where we synthesized substudies 1 and 2 and presented the findings to stakeholders invited from the 2 hospitals. The participants agreed on the most appropriate elements and topics in next-of-kin involvement in hospitals.

The study setting consists of 2 Norwegian university hospitals with their affiliated oncology departments. Both hospitals are affiliated with the same Regional Health Authority. The hospitals differ in size, number of employees, and budget, but the cancer departments are approximately the same size and are subject to the same national and regional policy documents (see details in Table 1).

**Table 1:** Local Context Descriptions With Key Figures

Local Context	Large City in Norway	Large City in Norway
Included hospitals	Hospital A	Hospital B
Size	University hospital Local hospital for 330 000 inhabitants	University hospital Local hospital for 420 000 inhabitants
Employees	7500	12 000
Budget	6.8 billion NOK	10.8 billion NOK
Cancer departments	Second largest regional cancer department with 2 cancer care wards, 2 outpatient clinics, and 1 radiotherapy unit	Main cancer department in the region with 2 cancer care wards, 1 outpatient clinic, and 1 radiotherapy unit

### The Norwegian Healthcare Context

Taxes fund the Norwegian healthcare system. All residents are covered by the National Insurance Scheme. The system is built on universal access and free choice of providers. Norway’s 4 Regional Health Authorities provide healthcare services within their district. The government has the financial oversight for all public hospitals.

Norway’s cancer registry reported 34 190 new cancer cases in 2018 and 283 984 people living with cancer.^28^ The incidence of cancer in Norway is higher than the average of the 36 Organisation for Economic Co-operation and Development countries (age-standardized rate ratio, 1.12), but the cancer mortality rate is lower (age-standardized rate ratio, 0.95).^29^

Under the Norwegian Patient and User Act (1999), the patient chooses the friend or family member who is the closest next- of-kin (§1.3b). The law does not specify any specific tasks or obligations for the next of kin in relation to the provision of healthcare services. The government has that responsibility in Norway; in other countries, there are stronger expectations that next of kin will take on a greater role in providing healthcare services.

### Theoretical Approach

#### ORGANIZING FOR QUALITY

**Figure 2:**
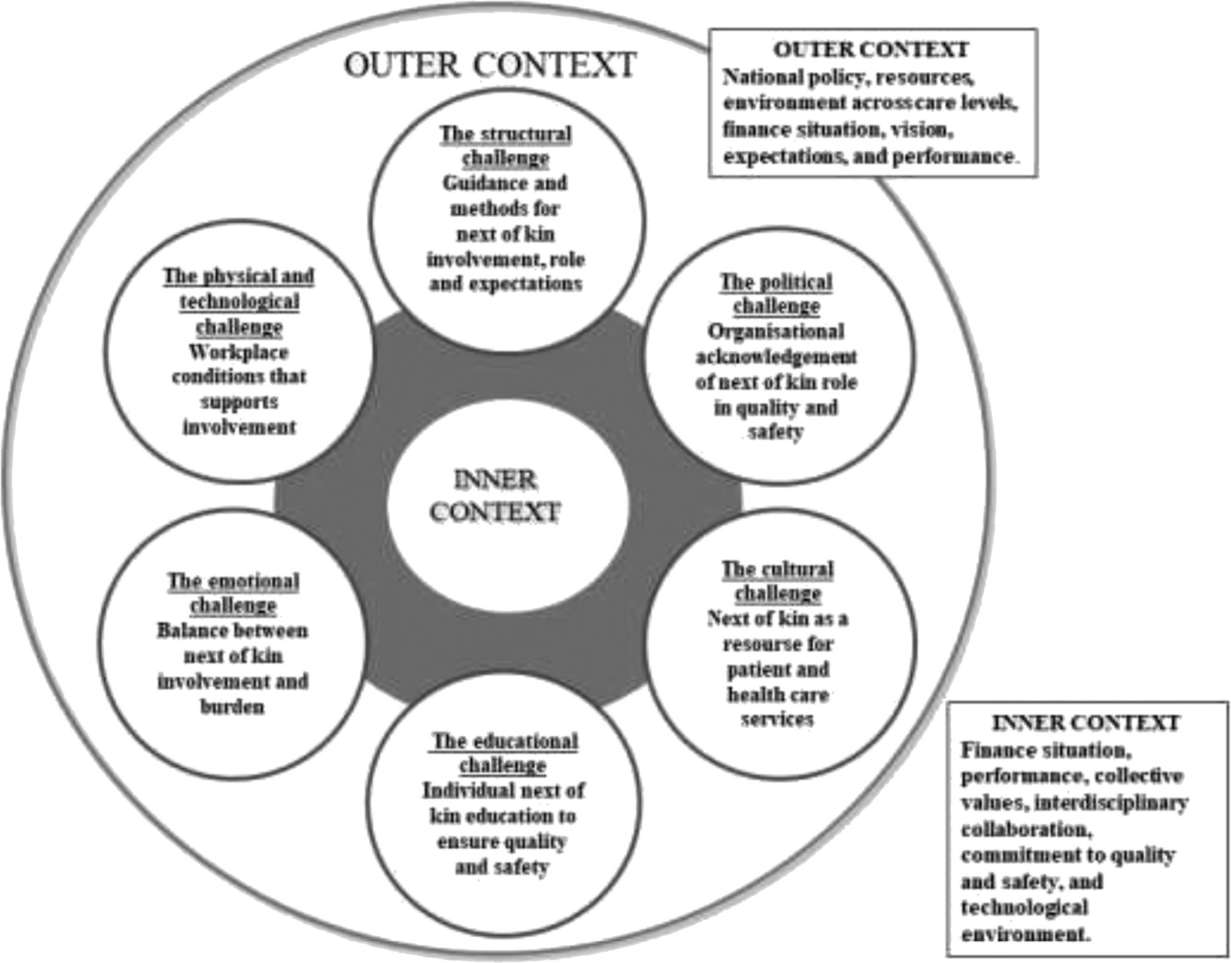
Revised framework model inspired by Bate and colleagues.^8,30^

The theoretical backdrop of this research project (Figure 1) is the Organizing for Quality (OQ) model developed by Bate and colleagues.^30^ The model focuses on 6 challenges that hospitals must meet (structural, political, cultural educational, emotional, physical and technological) as part of working on quality and safety in healthcare.^30^^(p169)^ The OQ model was developed based on international studies in leading European and American hospitals.^30–32^ It has also been tested and refined by studies in Norwegian hospitals.^33–35^ We apply a theoretical model in our research project to obtain the guidance to understand and investigate quality and safety processes in hospitals with a multilevel apporach.^36,37^ As a result of the first substudy (Figure 1), we suggested modifications to the OQ model. Figure 2 is built on the experience and views of leaders and healthcare professionals with next-of-kin involvement in the 2 hospitals. In Figure 2, we identified and elaborated on the 6 quality challenges and then added areas of key importance for next-of-kin involvement based on our findings to make it relevant for stakeholders in a clinical setting.^8^ Figure 2 is operationalized in this article into the next-of-kin involvement guide (Figure 5).

## Methods

The study design reported in this article is a consensus process inspired by the NGT. The NGT was developed by Delbecq and colleagues^25^ in 1975 and comprises 4 key elements: silent generation, round robin, clarification, and voting. All 4 elements are keys to arriving at a general agreement on a particular topic. The NGT is often used to explore stakeholders’ or consumers’ views, but the method can be modified for other purposes.^18^

**Figure 3:**
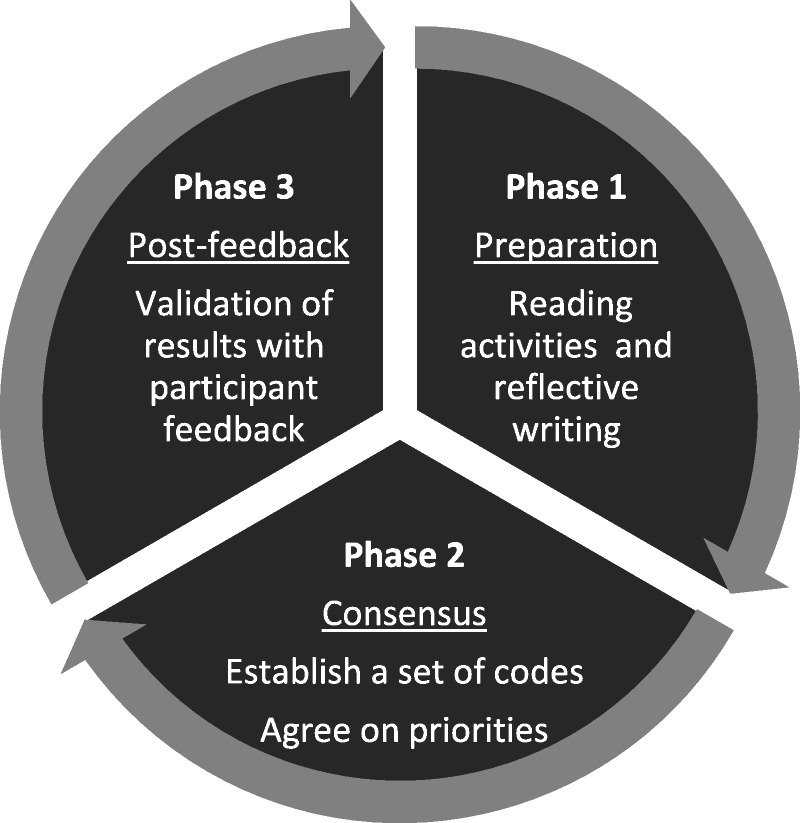
Overview of the 3-phase nominal group technique.

The modified NGT for this study was conducted in 3 phases to reach stakeholder agreement. Figure 3 is an overview of the process, consisting of preparation, consensus, and post-feedback, followed by validation of the results.

### Characteristics of Participants

Purposive sampling was used to identify healthcare professionals and next-of-kin representatives.^38^ Participation was voluntary and done in close collaboration with the 2 hospitals. Leaders from 7 inpatient and outpatient cancer care wards in the 2 hospitals participated in the recruitment of participants, among whom were leaders and multidisciplinary hospital staff. IJB contacted 1 coping center in both regions. The center, a meeting point for cancer patients and their representatives, offers courses, networking opportunities, and informal conversations. The 2 centers were asked if they would participate with 1 representative in the meeting. They also made contact with a local next-of-kin representative who was able to participate. For the consensus meeting, the Regional Health Authority appointed a regional next-of-kin representative. This representative was the only person who received compensation for this meeting in line with Regional Health Authority guidelines. Table 2 lists the panel participants for this study.

**Table 2:** Overview of Panel Participants

Participants in the Consensus Process	Number of Attendees
Local next-of-kin representatives	2
Regional next-of-kin representatives	1
Coping centers next-of-kin representatives	2
Hospital A—healthcare professionals	2 physicians and 6 oncology nurses
Hospital B—healthcare professionals	3 physicians and 7 oncology nurses
Gender of the participants	2 male and 18 female
Positions	5 managers and 15 healthcare professionals

### Overview of the Modified NGT

A consensus method, based on a modified NGT, was applied as a single 1-day meeting with 20 participants (5 next-of-kin representatives, 10 oncology nurses, and 5 physicians) from the 2 Norwegian university hospitals. The consensus meeting was supervised by a 5-member research team: 4 moderators (SW, GSB, BG, and IJB) and 1 nonparticipant observer (BF) who collected qualitative data on the nominal group processes during the 1-day meeting. This is recommended by Jones and Hunter.^17^ Observation notes were embedded in the analysis and used in the interpretation of the group process and results.

### Analysis

**Figure 4:**
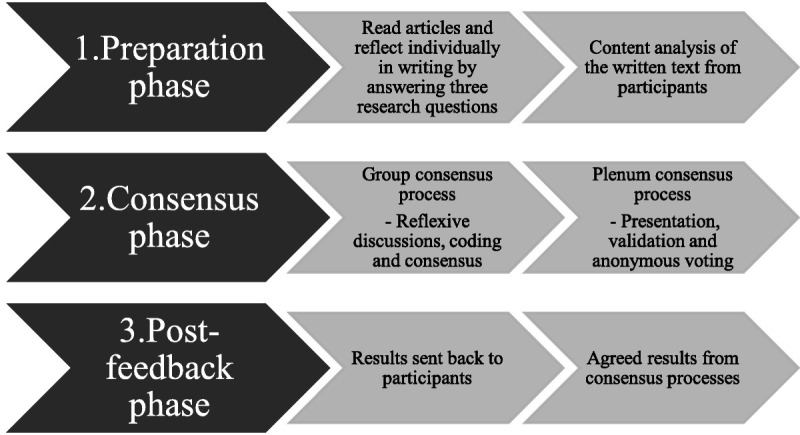
The modified nominal group technique.

The modified NGT developed for this study had 3 phases (Figure 3). The first phase was conducted by email, followed by a face-to-face meeting. The results were then emailed to the participants. The analysis process followed the 3 phases depicted in Figure 4.

#### PHASE 1: PREPARATION

In the first phase in the modified NGT, we had the participants engage in reading and reflective writing. One month before the meeting, we sent the participants 2 articles^8,27^ that described the results from the larger mixed-method project of which the consensus process is a part. We also asked the participants to reflect individually upon the topic “What is the role of next-of-kin for quality and safety in cancer care?” In addition, we asked them to respond in writing to the following questions that guided the consensus process:

1) What can we learn from next-of-kin experiences with hospital cancer care?
2) How can next-of-kin experiences be valued more systematically to improve cancer care quality and safety?
3) What methods or tools are appropriate for collecting experiences and for next-of-kin involvement (locally, regionally, nationally)?

The purpose of these assignments was to prepare each participant for the consensus process and to empower them to express themselves. Within 3 weeks, all the participants emailed a 1-page text to IJB with their thoughts and suggestions related to the research questions, earlier research findings, and their own experiences.

The research team led by IJB conducted a content analysis of the texts before the consensus meeting. The content analysis was inspired by Graneheim and colleagues.^39,40^ The analysis consisted of a 3-step characterization of the participants’ texts: (1) selecting meaning units, (2) condensing meaning units, and (3) defining subcategories and categories. The purpose of the content analysis was to identify categories and use these as an ice breaker to get all participants on the same page, before starting the consensus discussions in phase 2. An example of the content analysis can be found in Table 3.

**Table 3:** Example of the Content Analysis

Selecting Meaning Units	Condensing Meaning Units	Defining Subcategories	Defining Categories
“Next-of-kin experiences that are expressed can contribute to increased quality of healthcare. My experience is that collaboration with the next-of-kin in care and treatment of the cancer patient provides increased security in the patient’s coping with cancer and its treatment.”	Next-of-kin experiences can contribute to increased quality of healthcare. Collaboration with next-of-kin provides increased security in the patient’s coping with the disease and treatment.	Next-of-kin involvement is important for how well the patient is coping with disease and treatment.	Involvement of next-of-kin is important for coping with disease and treatment.

#### PHASE 2: CONSENSUS

The consensus meeting took place on a neutral arena that had no affiliation with any of the hospitals. Half of the participants had to travel by plane to attend the meeting in the city of one of the case hospitals. The meeting agenda is provided in the Appendix. The meeting started with a presentation by the participants, followed by a short introduction to the NGT and a summary of the results of previous substudies, and concluded with an overview of the content analysis on the emailed text from the participants. The participants learned about the views of leaders and healthcare professionals on next-of-kin involvement, the survey results from next-of-kin in the 2 hospitals, and the content analysis based on their initial reflections about these findings.

##### GROUP CONSENSUS—ESTABLISHING A COMMON SET OF CODES

After the introduction, we split the 20 participants into 2 groups to create a reflexive discussion, share experiences, generate new ideas, and establish a set of codes that the group could agree on for presentation in the following plenary session. Discussion questions were assigned for the first group session. Group 1 discussed these questions: What can we learn from next-of-kin experiences with hospital cancer care? How can next-of-kin experiences be valued more systematically to improve cancer care quality and safety? Group 2 discussed the question: What methods or tools are appropriate for collecting experiences for next-of-kin involvement (locally, regionally, nationally)? The 2 groups engaged in a consensus process led by moderators. The process was based on a reflexive discussion in which all suggestions were written on flip sheets, continued by a round-robin process until there were no more suggestions to discuss. Then the group and the moderators coded the suggestions by sorting and identifying common topics and suggestions. When the group reached consensus by agreeing on the codes, this session ended.

##### PLENARY CONSENSUS—AGREEING ON THE TOP 5 PRIORITIES

After the group sessions, we reunited the 2 groups in a plenary session. In the plenary session, all participants reached agreement on the codes set by the 2 smaller groups. The participants also completed an anonymous poll of the 5 initiatives that hospital cancer care services should prioritize when working on next-of-kin involvement. The plenary session was divided into 2 parts, with a plenary consensus process for each group’s research question. Each group presented the codes to the other and then discussed whether additional codes were needed. After the total group had reached agreement on the codes, we conducted anonymous voting on the 5 most important codes. Each participant manually submitted the votes to the research team. Both plenary consensus processes were completed in the same manner.

#### PHASE 3: POST-FEEDBACK

One week after the meeting, the participants received an email with the results of the anonymous voting session. We invited them to comment on the results. Only 1 participant responded, suggesting that we change the phrase “objective information” in priority 5 (Table 6) to “concrete information.” We embedded the revised wording in the code.

## Results

In the following, we present the results from the consensus meeting. Tables 4 and 5 show the codes from the group sessions, and Tables 6 and 7, the codes from the plenary session. We have incorporated the nonparticipant observers’ notes into the results presentation.

**Table 4:** Overview of Codes From Consensus 1: “What Can We Learn and How Can We Value”

Codes
Important for evaluating aid
Provides healthcare professionals with more objective or concrete information on the patient
Crucial for how well the patient handles the illness and treatment through the cancer care trajectory
Reveals areas where the help provided is not good enough
Next-of-kin who observe and interpret what happens to the patient are important, and they need to be trained in basic skills
Important throughout the cancer care trajectory. Next-of-kin have an eye for “the whole life”
Next-of-kin that are secure in their role can contribute to patient safety
Poor continuity of healthcare professionals creates unsafe next-of-kin
Healthcare professionals need more knowledge of next-of-kin involvement
Acknowledge the next-of-kin role as a coordination role that needs to be adjusted to individual needs
Next-of-kin experiences should be documented and systematized (user surveys, “heart sigh” book, next-of-kin notice in the documentation system)
Coherence between service levels (hospital and municipalities) with support from volunteer organizations
Be aware of those patients who do not have a next-of-kin
System improvement that uses next-of-kin evaluation as a measure (user surveys)
Double loop learning with respond to service users

**Table 5:** Overview of Codes From Consensus 2: “Methods and Tools for Collecting Experiences”

Codes
Technology (apps, documentation, admission forms)
Economy (travel expenses, time off work, consultations, diagnose related groups’ effort-based funding, social rights as a next-of-kin)
Involvement in patient care (clarification of roles, different phases of the trajectory (curative or palliative), standardization of involvement in different parts of the trajectory, documentation)
Needs clarification/information in the summon letter and in different phases (expectations, resources, wishes and needs, information in summon letter and different phases, checklist on needs at discharge, information)
Interaction (learning and coping centers in the municipalities)
Information (to next-of-kin, learning and coping)
Training of healthcare professionals (ethics, how, methods)
One appointed healthcare professional for the next-of-kin
User participation with special focus on the next-of-kin perspective

**Table 6:** Top 5 Priorities Consensus 1: “What Can We Learn and How Can We Value?”

What can we learn from next-of-kin experiences with hospital cancer care? How can next-of-kin experiences be valued more systematically to improve the quality and safety of cancer care?	
1	Next-of-kin experiences should be documented and systematized (user surveys, “heart sigh” book, next-of-kin notice in the documentation system).
2	Next-of-kin who are secure in their role can contribute to patient safety.
3	System improvement that uses next-of-kin evaluation as a measure (user surveys).
4	Reveals areas where the help provided is not good enough.
5	Important for evaluating aid.
5	Provides healthcare professionals with more objective or concrete information on the patient.
5	Crucial for how well the patient handles the illness and treatment through the cancer care trajectory.
5	Next-of-kin who observe and interpret what happens to the patient are important, and they need to be trained in basic skills.

**Table 7:** Top 5 Priorities in Consensus 2: “Methods and Tools for Collecting Experiences”

What methods or tools are appropriate for collecting experiences and for involvement of next-of-kin (locally, regionally, nationally)?	
1	Involvement in patient care (clarification of roles, different phases of the trajectory [curative or palliative], standardization of involvement in different parts of the trajectory, documentation)
2	Interaction (learning and coping centers in the municipalities)
3	Information (to next-of-kin, learning and coping centers)
4	Training of healthcare professionals (ethics, how, methods)
5	Technology (apps, documentation, admission forms)

### Group Consensus Results

#### GROUP CONSENSUS 1: “WHAT CAN WE LEARN AND HOW CAN WE VALUE NEXT-OF-KIN INVOLVEMENT?”

Table 4 summarizes the codes from the group discussion process in response to the questions: What can we learn from next-of-kin experiences with hospital cancer care? How can next-of-kin experiences be valued more systematically to improve the quality and safety of cancer care? There was a good atmosphere in this group. According to the nonparticipant observers’ notes, all the participants were engaged in contributing to the process. The next-of-kin representatives were courageous and added important input. The physicians were initially a little reticent, but according to the observation notes, all participants were seen by the moderators in this group. The results acknowledged the next-of-kin’s central role in patient care as the most important learning dimension for next-of-kin involvement. Participants highlighted that next-of-kin possess essential information about the patient, are central to care coordination, and give valuable feedback about how patients respond to the treatment.

#### GROUP CONSENSUS 2: METHODS AND TOOLS FOR COLLECTING EXPERIENCES

Table 5 gives an overview of the codes from the group discussion process with respect to this question: What methods or tools are appropriate for collecting experiences and for involvement of next-of-kin (locally, regionally, nationally)?

According to the nonparticipant observer’s notes, there was very good participation and engagement in this group. Moreover, all participants were seen by the moderators in this group, and the group progressed with the help of the moderators. The group seemed to struggle with coding the discussion moments and needed the moderators’ assistance. Engagement declined slightly in the coding phase. However, the group members remained engaged and shared their views on the topic of the session. The results focused on standardization of involvement in different parts of the cancer care trajectory as the most important tools and methods to integrate into a guide. They suggested use of apps, a checklist, and the medical record document and improve involvement.

### Plenary Consensus Results

#### AGREEING ON TOP 5 PRIORITIES

Tables 6 and 7 give an overview of the results of the anonymous voting on the 2 top 5 priorities for hospitals’ cancer care services to address. The top 5 priorities are meant for service development use to support next-of-kin involvement in cancer care, especially in relation to (1) learning and information and (2) recommendation of methods to promote involvement in practice.

According to the nonparticipant observer’s notes, there was less engagement in the plenary process than in the 2 previous separate group discussions. Even if it was a more challenging plenary process, it generated discussion and new insights.

### Evaluation of the Method and the Meeting

At the end of the day, an evaluation session allowed the participants to share their views on the consensus meeting. The group said that it had been very useful for them to have come to the meeting prepared. The group highlighted that the meeting had been a good arena to explore and discuss next-of-kin involvement. They also noted that they felt safe sharing their opinions and speaking their minds. One next-of-kin representative thought that the inclusion of more next-of-kin representatives in the meeting could have contributed more input.

## Discussion

### Developing Key Concepts for Next-of-Kin Involvement in Hospital Cancer Care

In this article, we presented the results from a consensus process with the purpose of identifying key topics and elements that should be included in a next-of-kin involvement guide for quality and safety in hospital cancer care. The purpose of the process was to describe and suggest changes for next-of-kin involvement practice in hospital cancer care, but it can also be relevant for other healthcare services or decision-making support bodies. The top 5 priorities in this study show that next-of-kin are considered key stakeholders in keeping the patient safe. The stakeholder groups emphasized that, first, it is important for cancer care services to start developing systems for the systematization and documentation of next-of-kin experiences for further use. An example could be by integrating data on next-of-kin experiences, for instance, in user surveys.^15^

Second, the panel agreed that hospital cancer care needs to recognize and change service in a direction that formally integrates and uses next-of-kin experiences in service improvement at the micro level. Moreover, there was consensus in terms of personalized next-of-kin training and support to prepare them for the challenges and care tasks that they will perform. There was agreement that treating next-of-kin as an equal part of the patient’s medical care team is a prerequisite for sound next-of-kin involvement. Our findings are in line with other studies highlighting next-of-kin as an underused resource, for evaluating aid and providing healthcare professionals with more objective information on the patient’s condition.^12,13,41–45^

Another important message from our consensus process is that hospital cancer care should become more aware of how to use next-of-kin experiences because of its potential impact on how well the patient handles treatment and care. In other words, next-of-kin involvement in cancer care is important for patient outcome and should be a higher priority in future practice. This message echoes other studies that highlighted the important role of next-of-kin involvement in healthcare.^5,6,10,13,44,45^

### Organizing for Quality and Safety: A Next-of-Kin Involvement Guide

There is a constant call for theory development in research and for incorporating theory into everyday practice in healthcare organizations.^46^ Our project responds to this call and builds on Bate and colleagues’^8,30^ conceptualization of quality and safety in healthcare. The project is also in line with experience-based co-design^47^ by combining participatory design and user experiences in developing a guide to improve cancer care services. Co-design in this study has required the participation of multidisciplinary healthcare professionals within cancer care and next-of-kin representatives from 2 university hospitals to share and reflect on their experiences to identify priorities for implementation of change.^48,49^

**Figure 5:**
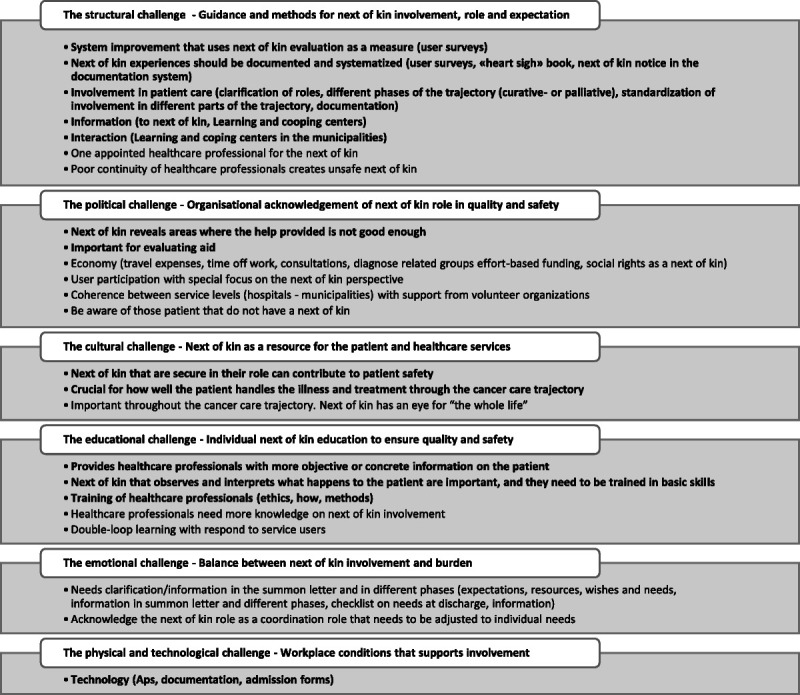
Organizing for quality and safety: a next-of-kin involvement guide.

As previously mentioned, we apply the OQ as our theoretical backdrop, which we have modified to fit next-of-kin involvement in cancer care (see Figure 2). We will now present the next-of-kin involvement guide (Figure 5) that encompasses, develops and operationalizes Figure 2 with the results from the consensus process. We want to give the model (Figure 2) a broader empirical foundation, one that incorporates a multistakeholder approach. The main purpose is, however, to convert the model into a practical tool with direct connection to both theory and knowledge-based adaptations derived from stakeholder involvement and the consensus process. Until now, this has been lacking in the research literature.^19–21,50^

Figure 5 illustrates the next-of-kin involvement guide. The guide is a result of merging the framework model (Figure 2) and the results from the consensus process (Tables 4 and 5). Through this merger, we have developed a guidance tool for hospital cancer care services by translating theory into practice with suggestions on where to start making changes to explore and support next-of-kin involvement. The stakeholder groups agreed on top 5 priorities in each of the 2 consensus sessions. These priorities are bolded in the figure; however, the stakeholder groups did not state that the additional codes had a lower priority. Therefore, we embedded all suggestions in the figure and grouped them under the 6 quality challenges.

The guide can be used in either as a guide with suggestions and priorities or as a reflexive tool for improvement efforts in the organization. The latter approach has been adapted and explored with the OQ model,^31,51^ in the Norwegian primary care context,^37,52^ and in international studies.^53–55^

### Implications for Practice, Research and Education

Next-of-kin involvement in healthcare services is complex. Like Bell and colleagues,^16^ we contend that decision and actions within this area should be based on a multistakeholder approach where the perspectives of all stakeholders are heard and integrated. This study adds to the knowledge of how to create an arena for hospitals to share ideas and learn from each other and from involved next-of-kin stakeholders. The reflexive space established through the consensus process presented in this article brings attention to practical values and challenges of next-of-kin involvement, which can inform everyday practice in hospitals. A key rationale for reflexive practice is bringing together stakeholders with the ability to engage in the cocreation of knowledge that supports organizational learning to reach a higher level of understanding.^14,56–58^ This study explains how the consensus method can be used for different purposes in hospitals, such as the development of internal guidelines, evaluation of performance, change management, interventions, compliance, and communication between disciplines or institutions.

At the same time, there is potential to identify priority topics for research and practice improvement by using consensus methods. This has been demonstrated in other studies^59,60^ that have set research priorities with the use of a consensus design. For educational purposes, the methodological approach can target future strategic directions with input from stakeholders involved in the specific areas or questions of interest such as cancer care, diabetes, and pediatrics. However, how successful this translation of knowledge and learning turns out to be, depends on how healthcare professionals value research, develop knowledge and use this proactively for innovation.^61^

Further studies and practical testing of the next-of-kin involvement guide are needed. Future evaluations should focus on how relevant and applicable the guide (Figure 5) is perceived by the hospitals and the clinical staff and how they respond to and modify their practice accordingly.^50,62^

We envision future testing of the guide for diverse purposes. Nursing staff on cancer wards could use it to reflect on current practice and discuss potential changes. It could also be tested in multidisciplinary teams of nurses, doctors, and managers in cancer care departments to assess structures, culture, and methods in use and what could be changed to strengthen next-of-kin involvement. We envision, for example, dialogue cafes in which patients, next-of-kin, and healthcare professionals use the guide as a basis of discussion.

### Strengths and Limitations

This study has both strengths and limitations. First, the consensus meeting was a face-to-face 1-day meeting. Because of the extensive consensus processes, this meeting could have benefited from being extended by 1 day. However, funding constraints made this impossible. All participants from one of the hospitals had to travel by plane for this meeting, and a 1-day extension would have increased the cost and kept healthcare professionals out of clinical work for an additional day. Consequently, recruiting healthcare professionals for a 2-day meeting would have been more difficult.

A second limitation was sample size and representativeness of care providers. Healthcare professionals were the largest group in the interdiciplinary team of care providers, and an increased number of user representatives might have produced an even better understanding of the 6 challenges mentioned in the involvement guide. We mixed the groups with healthcare professionals and next-of-kin representatives to try to create consensus across diciplines and stakeholder groups with potentially different perspectives. This was done in line with the multistakeholder approach in this study. We have tried our best to meet ethical standards by having each participant prepare for the meeting by reading, reflecting and writing; to engage in the meeting through the introduction of research results and content analysis; by engaging a nonparticipant observer (observing power in the groups); and by asking the moderators to be aware of the potential risk of uneven power relations in the groups. However, we cannot rule out the potential of participants who did not dare to speak up in the mixed groups.

Third, there is a possibility that asking the participants to read and reflect on earlier published papers might have affected their views on the topic and could, in that sense, be a limitation. However, this could also be one of the study’s strengths. This is a key step in the modified NGT (Figure 4) and a way to retrieve and embed feedback to ensure stakeholder involvement in the research project.

## Conclusions

In this article, we have described a nominal group consensus technique conducted with representatives from cancer departments in 2 Norwegian university hospitals. We included next-of-kin representatives and healthcare professionals within hospital cancer care. During the process, they identified key topics and elements in next-of-kin involvement. Based on the results, we developed a guide for next-of-kin involvement in cancer care. The guide (Figure 4) is created to support hospitals and has the potential to increase attention to and overcome challenges in next-of-kin involvement. Moreover, it emphasizes the role of next-of-kin and their importance for quality and safety in cancer care. Service providers can use the guide to develop and improve next-of-kin involvement practice or as a reflexive tool for organizational improvement. However, for future research, the guide needs additional empirical testing and refinement.

## Appendix Agenda of the meeting.

**Table:**

08:30-09:00 am	Registration
09:00-10:10 am	Introduction
10:10-10:30 am	Break
10:30 am-12:00 noon	Group process (2 groups)
12:00 noon-1:00 pm	Lunch
1:00-2:15 pm	Plenary process 1
2:15-2:35 pm	Break
2:35-3:40 pm	Plenary process 2
3:40-4:00 pm	Summary
